# Endocrine Disorders of Calcium Signaling in Children: Neuroendocrine Crosstalk and Clinical Implications

**DOI:** 10.3390/cells15020140

**Published:** 2026-01-13

**Authors:** Roberto Paparella, Francesca Pastore, Lavinia Marchetti, Arianna Bei, Irene Bernabei, Norma Iafrate, Vittorio Maglione, Marcello Niceta, Anna Zambrano, Mauro Celli, Marco Fiore, Ida Pucarelli, Luigi Tarani

**Affiliations:** 1 Department of Maternal Infantile and Urological Sciences, Sapienza University of Rome, 00185 Rome, Italy; 2 Molecular Genetics and Functional Genomics, Ospedale Pediatrico Bambino Gesù, IRCCS, 00165 Rome, Italy; 3 Institute of Biochemistry and Cell Biology (IBBC-CNR), c/o Department of Sensory Organs, Sapienza University of Rome, 00185 Rome, Italy

**Keywords:** calcium signaling, calcium channelopathies, pediatric neuroendocrine disorders, neurodevelopment, calcium-targeted therapeutics

## Abstract

Calcium ions (Ca^2+^) serve as universal second messengers regulating endocrine, neuronal, and metabolic processes. In children and adolescents, tight calcium signaling control is crucial for growth, hormone homeostasis, neuromuscular function, and neurodevelopment. Disruptions in Ca^2+^-dependent pathways—whether genetic, metabolic, or acquired—underlie a spectrum of pediatric endocrine diseases often presenting with neurological manifestations This review summarizes calcium’s roles in hormone secretion, parathyroid and vitamin D metabolism, and neuronal excitability, and discusses monogenic and metabolic disorders affecting calcium sensing and signaling, including *CASR*, *GNA11*, *AP2S1*, *STIM1*, and *ORAI1* mutations. Diagnostic challenges, therapeutic strategies, and future directions for precision medicine in pediatric neuroendocrinology are highlighted, emphasizing early recognition and improved clinical outcomes.

## 1. Introduction

Calcium acts as a universal second messenger in both endocrine and neuronal physiology, coordinating a broad range of cellular processes through tightly regulated signaling mechanisms. In neurons, calcium is essential for neurotransmitter release, synaptic plasticity, and membrane excitability. Following neuronal depolarization, voltage-gated calcium channels (VGCCs) open, allowing calcium ion (Ca^2+^) influx, which triggers synaptic vesicle fusion and neurotransmitter exocytosis. Intracellular calcium further regulates gene expression, neuronal growth, differentiation, and survival [1]. The precise control of calcium dynamics—mediated by buffering proteins, membrane pumps and exchangers, and intracellular stores such as the endoplasmic reticulum (ER) and mitochondria—is critical for effective neuronal function and plasticity. Disruptions in calcium homeostasis contribute to neurodegenerative and neurodevelopmental disorders, including Alzheimer’s disease, Parkinson’s disease, epilepsy, migraine, autism spectrum disorder, and attention deficit hyperactivity disorder (ADHD) [1,2,3].

In endocrine cells, calcium is essential for stimulus–secretion and stimulus–transcription coupling. Activation of VGCCs or G protein-coupled receptors generates Ca^2+^ oscillations that drive hormone exocytosis. The multiplicity of calcium entry and release mechanisms, including intracellular stores and gap junction communication, ensures finely tuned endocrine responses to physiological stimuli [4,5,6,7,8,9]. For example, in pancreatic beta cells, glucose-induced depolarization leads to Ca^2+^ influx, triggering insulin secretion [9]. Similar calcium-dependent mechanisms regulate hormone release in the parathyroid and adrenal glands. Overall, calcium functions as a master integrator of rapid and coordinated endocrine and neuronal responses, maintaining cellular and systemic homeostasis.

Historically, bone and mineral metabolism were viewed primarily in relation to structural support, locomotion, and the maintenance of calcium and phosphate homeostasis. Bone was regarded as a passive mineral reservoir and a site of hematopoiesis, with little systemic endocrine relevance [10,11]. Calcium itself was conceptualized largely as a static structural ion essential for bone strength and extracellular mineral balance. This reductionist perspective dominated the field for much of the twentieth century. However, beginning in the mid-twentieth century, calcium was increasingly recognized as a dynamic intracellular signaling molecule, expanding its physiological profile beyond structural functions to incorporate roles in systemic regulation and intercellular communication.

More recently, bone has emerged as a true endocrine organ. Osteoblasts and osteocytes secrete hormones such as osteocalcin and fibroblast growth factor-23 (FGF-23), which regulate energy metabolism, glucose homeostasis, male fertility, and cardiovascular function [10,12,13,14,15]. These bone-derived hormones reveal a bidirectional communication network linking bone to the pancreas, kidney, adipose tissue, and brain, integrating skeletal biology into complex multisystem physiology [15,16,17]. This conceptual shift reflects broader developments in modern medicine, which increasingly view organs as interconnected components of dynamic signaling networks influencing health and disease [8].

Calcium signaling plays a pivotal role in neuroendocrine function by acting as the final common intracellular mediator that couples membrane excitability and receptor activation to hormone and neurotransmitter secretion. In neuroendocrine cells, transient or sustained increases in intracellular Ca^2+^ directly regulate vesicle fusion, gene transcription, and long-term cellular adaptation, thereby coordinating endocrine outputs with neuronal activity. During childhood, when neuronal circuits and endocrine axes are undergoing dynamic maturation, precise regulation of calcium signaling is particularly critical. Disruptions in calcium sensing or intracellular Ca^2+^ handling therefore have the potential to simultaneously impair hormonal homeostasis and neurodevelopmental processes, explaining the frequent coexistence of endocrine and neurological manifestations in pediatric calcium signaling disorders.

Pediatric neuroendocrine disorders related to calcium metabolism arise mainly from abnormalities in the hormonal regulation of calcium involving parathyroid hormone (PTH), vitamin D, the calcium-sensing receptor (CaSR), and, in some cases, FGF-23. These disorders may manifest as hypocalcemia or hypercalcemia with neurological and systemic symptoms [18,19,20,21]. Activating mutations in *CASR* cause autosomal dominant hypocalcemia type 1 (ADH1), whereas inactivating mutations lead to familial hypocalciuric hypercalcemia (FHH) or neonatal severe hyperparathyroidism (NSHPT) [22,23,24]. Excess PTH secretion, as seen in primary hyperparathyroidism or multiple endocrine neoplasia syndromes, results in hypercalcemia, while deficiency—such as hypoparathyroidism associated with 22q11.2 deletion syndrome—causes hypocalcemia [18,25,26]. Disorders of vitamin D synthesis or receptor function result in hypocalcemic rickets, whereas excessive vitamin D exposure leads to hypercalcemia [24,27]. Although FGF-23 primarily regulates phosphate metabolism, its activity may indirectly influence calcium balance [28]. Diagnosis relies on biochemical evaluation of calcium, phosphate, PTH, vitamin D, and magnesium levels, with genetic testing when indicated [18,19,26]. Treatment varies by etiology and may include calcium and vitamin D supplementation, hydration and antiresorptive therapy for hypercalcemia, or targeted interventions for genetic and syndromic forms [18,24,25].

Importantly, calcium signaling pathways exhibit marked developmental specificity. During fetal life, infancy, and childhood, calcium-dependent processes regulate not only endocrine secretion but also neuronal migration, synaptogenesis, myelination, and circuit maturation. Consequently, disturbances in calcium sensing or signaling during critical developmental windows may lead to long-lasting neuroendocrine and neurocognitive consequences that differ substantially from those observed in adult-onset disorders. This developmental vulnerability provides a strong rationale for a pediatric-focused analysis of calcium signaling disorders.

### Review Methodology and Scope

This article is conceived as a narrative review with a structured, hypothesis-driven approach rather than a fully systematic analysis. The literature was identified through searches of major biomedical databases, including PubMed/MEDLINE and Scopus. The time frame primarily encompassed publications from the last two decades, with the inclusion of earlier landmark studies when relevant to calcium signaling physiology.

Article selection was guided by clinical and translational relevance, with particular emphasis on pediatric populations, genetically determined disorders of calcium sensing and signaling, and conditions characterized by combined endocrine and neurological manifestations. Both experimental and clinical studies were considered to integrate molecular mechanisms with developmental and clinical phenotypes. This approach was chosen to provide a comprehensive and conceptually integrated overview of calcium signaling disorders in children, rather than an exhaustive quantitative synthesis.

We aim to illustrate how calcium physiology extends beyond classical mineral homeostasis to a complex web of inter-organ communication involving the brain, endocrine tissues, and bone as an endocrine organ. By connecting cellular physiology—including channels, pumps, regulatory proteins, and hormones—to clinical phenotypes such as hypo- and hypercalcemia, we highlight the interplay of genetic, hormonal, and environmental factors underlying pediatric neuroendocrine calcium disorders. Finally, we discuss therapeutic implications, including current management strategies and emerging treatments targeting CaSR or vitamin D metabolism, and outline future directions toward precision medicine based on a deeper molecular understanding of calcium signaling networks.

## 2. Physiology of Calcium Signaling in Endocrine and Nervous Systems

### 2.1. Cellular Mechanisms of Calcium Signaling

The dynamics of intracellular calcium ions are regulated by an integrated system of channels, pumps, and sensors that coordinate Ca^2+^ movement between cellular compartments and the cytosol, enabling the transduction of physiological signals. The principal intracellular calcium-release channels include inositol 1,4,5-trisphosphate receptors (IP_3_Rs) and ryanodine receptors (RyRs), located on the membranes of the ER and the sarcoplasmic reticulum. These channels rapidly mobilize Ca^2+^ in response to diverse stimuli, amplifying intracellular signals through mechanisms such as calcium-induced calcium release and the propagation of calcium waves [29,30]. In striated muscle, functional coupling between voltage-operated channels and RyRs is essential for excitation–contraction coupling [31].

Reuptake and extrusion mechanisms ensure the restoration of basal cytosolic calcium levels. The sarco/ER Ca^2+^-ATPase (SERCA) pumps Ca^2+^ back into the ER/sarcoplasmic reticulum, whereas plasma membrane Ca^2+^-ATPases (PMCAs) and Na^+^/Ca^2+^ exchangers (NCXs) extrude Ca^2+^ from the cell, maintaining low cytosolic Ca^2+^ concentrations and preventing calcium overload [32,33]. Calcium sensors such as calmodulin and stromal interaction molecule 1 (STIM1) detect fluctuations in intracellular Ca^2+^ levels. Upon ER Ca^2+^ depletion, STIM1 undergoes conformational rearrangements that enable the activation of ORAI1 channels in the plasma membrane, generating extracellular Ca^2+^ influx through the store-operated calcium entry (SOCE) pathway [34,35]. This interaction produces calcium release-activated calcium (CRAC) currents, which can oscillate in synchrony with cytosolic Ca^2+^ dynamics [36].

Mitochondria act as dynamic calcium buffers and shape intracellular calcium signals by rapidly taking up Ca^2+^ through the mitochondrial calcium uniporter, thereby modulating the amplitude, frequency, and duration of Ca^2+^ transients [37,38]. Through these combined actions, the coordinated function of channels, pumps, and sensors ensures precise spatial and temporal modulation of intracellular Ca^2+^, which is fundamental for processes including muscle contraction, hormone secretion, gene transcription, and cell survival [32,33,37]. Mitochondrial calcium handling is closely linked to oxidative stress pathways, which can further modulate calcium signaling and neuronal vulnerability. Disruption of this interplay has been implicated in pediatric genetic and endocrine disorders characterized by neurodevelopmental impairment [39,40,41,42].

Intracellular Ca^2+^ signaling also plays a central role in the regulation of endocrine secretion. In parathyroid cells, Ca^2+^ exerts an inhibitory effect on PTH release: increased cytosolic Ca^2+^ suppresses secretion, whereas reduced Ca^2+^ levels stimulate it, a mechanism mediated by CaSR and calcium-activated potassium channels that preserve systemic calcium homeostasis [43]. In pancreatic β-cells, glucose metabolism leads to membrane depolarization and Ca^2+^ influx through VGCCs, which acts as the final trigger for insulin granule exocytosis [44,45]. In pituitary somatotrophs, growth hormone secretion is regulated by Ca^2+^ entry in response to hypothalamic signals. Growth hormone–releasing hormone stimulates, while somatostatin inhibits, growth hormone release by acting on G-protein-coupled receptors that modulate membrane potential, second-messenger pathways, and intracellular Ca^2+^ concentrations [5,8,46,47]. Across all these endocrine systems, Ca^2+^ pumps and sensors such as calmodulin and synaptotagmin ensure temporal precision and adequate amplitude of Ca^2+^ signals, tightly coupling calcium dynamics to hormone secretion [4].

### 2.2. Calcium-Sensing and Receptor Systems

CaSR is a key regulator of extracellular Ca^2+^ homeostasis and orchestrates multiple intracellular signaling pathways in response to changes in extracellular calcium levels. Upon activation, CaSR engages several G-protein-coupled pathways [48]. Through Gq/11, it stimulates the phosphoinositide cascade, leading to the generation of inositol trisphosphate (IP_3_) and diacylglycerol (DAG), which promotes Ca^2+^ release from the ER. Signaling through Gi/o reduces cyclic adenosine monophosphate (cAMP) production, while activation of G12/13 contributes to additional downstream cellular responses. Together, these pathways converge to modulate intracellular Ca^2+^ concentrations and the Ca^2+^/cAMP axis, producing cell-type-specific physiological outputs [49,50].

Multiple channels participate in fine control of Ca^2+^ entry. Among these, transient receptor potential vanilloid 6 (TRPV6) is a highly calcium-selective channel essential for intestinal and renal Ca^2+^ absorption. TRPV6 activity is regulated by intracellular Ca^2+^ and by phosphoinositides, particularly phosphatidylinositol 4,5-bisphosphate (PIP_2_), and can be modulated by CaSR-dependent signaling, establishing a functional connection between extracellular calcium sensing and transmembrane Ca^2+^ transport [51,52,53].

**Figure 1:**
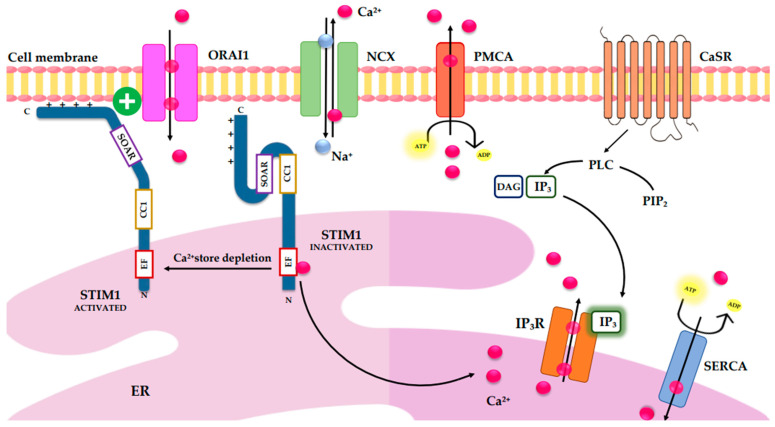
Overview of store-operated calcium entry (SOCE). Activation of CaSR (PLC-coupled receptors) by agonists triggers subsequent IP_3_-mediated calcium release, resulting in depletion of calcium from the ER. Pumps, such as SERCA, recapture Ca^2+^ into the ER, while PMCA and NCXs expel Ca^2+^ from the cell, maintaining low cytosolic concentrations. Calcium sensors, including STIM1, detect variations in Ca^2+^ levels and control the activation of channels like ORAI1, enabling extracellular Ca^2+^ entry through the store-operated calcium entry mechanism (SOCE). STIM1 undergoes a conformational alteration that extends its C-terminal region toward the plasma membrane, where it interacts with ORAI1 channels and generates currents through CRAC channels. Alterations in key components of the SOCE machinery have been directly implicated in human disease. Loss-of-function mutations in *STIM1* or *ORAI1* genes cause severe combined immunodeficiency-like phenotypes with immune dysfunction, myopathy, and ectodermal abnormalities, whereas gain-of-function variants lead to Stormorken syndrome and tubular aggregate myopathy. Dysregulation of CaSR-mediated signaling upstream of SOCE contributes to calcium-sensing disorders such as autosomal dominant hypocalcemia and familial hypocalciuric hypercalcemia, highlighting the clinical relevance of the CaSR–PLC–IP_3_–STIM1–ORAI1 axis discussed in this review. Abbreviations: ADP, adenosine diphosphate; ATP, adenosine triphosphate; Ca^2+^, calcium; CaSR, calcium-sensing receptor; CC1, coiled-coil 1; DAG, diacylglycerol; ER, endoplasmic reticulum; IP_3_, inositol trisphosphate; IP_3_R, inositol 1,4,5-trisphosphate receptor; Na^+^, sodium; NCX, Na^+^/Ca^2+^ exchanger; PIP_2_, phosphatidylinositol 4,5-bisphosphate; PLC, phospholipase C; PMCA, plasma membrane Ca^2+^-ATPase; SERCA, sarco/ER Ca^2+^-ATPase; SOAR, STIM1 ORAI-activating region; STIM1, stromal interaction molecule 1.

A central component of calcium homeostasis is the STIM1/ORAI1 system, which mediates store-operated calcium entry (SOCE) [Figure 1]. STIM1 acts as an ER Ca^2+^ sensor: upon depletion of luminal Ca^2+^—often triggered by IP_3_-mediated release—STIM1 undergoes structural rearrangements and migrates toward ER–plasma membrane junctions, where it recruits and activates ORAI1 channels. This interaction enables a controlled influx of extracellular Ca^2+^, restoring ER calcium stores and sustaining prolonged cytosolic Ca^2+^ signaling [54,55,56].

STIM1 also exerts inhibitory control over VGCCs, thereby integrating store-dependent and voltage-dependent Ca^2+^ entry. This cross-regulation ensures that Ca^2+^ influx is precisely coordinated across different physiological contexts [57,58]. VGCCs themselves constitute an additional mechanism of calcium entry, especially in excitable cells, where they are fundamental for muscle contraction, neurotransmitter release, hormone secretion, and the activity-dependent modulation of gene expression [59,60,61]. Their function is influenced not only by cytosolic Ca^2+^ but also by cAMP through protein kinase A-mediated phosphorylation, establishing a bidirectional interaction between electrical excitability and second-messenger pathways [62,63].

Overall, CaSR, TRPV6, STIM1/ORAI1, and VGCCs constitute a finely coordinated regulatory network in which Ca^2+^ entry and interactions with the cAMP, mitogen-activated protein kinase, and phosphoinositide pathways allow precise control of calcium homeostasis and a wide spectrum of cellular functions.

### 2.3. Neuroendocrine Integration

A series of shared pathways operates in both endocrine and neuronal cells, in which Ca^2+^ functions as the principal mediator of signal transduction. In both cell types, membrane depolarization is converted into a rapid rise in cytosolic Ca^2+^ through the activation of VGCCs. This increase represents the proximal signal that triggers exocytosis of neurotransmitters in neurons and of hormones in endocrine cells [4,8,64,65]. Although the core mechanism of excitation–secretion coupling is conserved, the submembrane architecture differs markedly: in neuroendocrine cells, the greater distance between VGCCs and secretory vesicles produces a less tightly coupled configuration (“loose coupling”), requiring high-frequency activity—such as burst firing—to generate Ca^2+^ microdomains sufficient to support secretion [65].

Despite sharing fundamental calcium-dependent mechanisms, neurons and endocrine cells differ markedly in the spatial and temporal organization of Ca^2+^ signals. In presynaptic neuronal terminals, calcium entry through tightly clustered VGCCs generates highly localized, short-lived Ca^2+^ nanodomains that trigger rapid, millisecond-scale neurotransmitter release. This process requires precise spatial coupling between calcium channels and synaptic vesicles, ensuring temporal fidelity and fast synaptic transmission.

In contrast, endocrine cells, such as parathyroid chief cells, rely on slower and more spatially diffuse Ca^2+^ signals. Calcium influx through VGCCs and store-operated calcium entry is integrated with calcium release from intracellular stores, producing sustained or oscillatory Ca^2+^ elevations that regulate hormone synthesis and secretion over seconds to minutes. This looser spatial coupling allows endocrine cells to decode calcium signal frequency and duration rather than peak amplitude, thereby translating systemic cues into graded hormonal outputs.

These differences underscore the concept of neuroendocrine crosstalk: while neurons prioritize speed and spatial precision, endocrine cells emphasize signal integration and temporal summation. Perturbations of calcium signaling pathways therefore have distinct but converging consequences in the nervous and endocrine systems, explaining the frequent coexistence of neurological and hormonal phenotypes in pediatric calcium signaling disorders.

In addition to transmembrane influx, cytosolic Ca^2+^ is further shaped by mobilization from intracellular stores in the ER through inositol trisphosphate receptors (IP_3_Rs) and, to a variable extent, RyRs [3,64,66]. The combined contribution of extracellular Ca^2+^ entry and intracellular store release generates oscillatory or sustained signals that regulate membrane excitability, potentiate synaptic transmission, and modulate endocrine secretion. The temporal and spatial characteristics of these signals are refined by calcium-buffering proteins and by mitochondrial uptake, which regulate the amplitude, duration, and propagation of Ca^2+^ microdomains [3].

A further level of integration arises from hormonal feedback mechanisms. G-protein-coupled receptors, activated by circulating hormones or autocrine and paracrine signals, modulate membrane excitability and intracellular Ca^2+^ handling through pathways such as adenylyl cyclase–cAMP/protein kinase A and phospholipase C–IP_3_/DAG [8,66]. Excitatory pathways can enhance Ca^2+^ influx by phosphorylating ion channels or regulating potassium channels, whereas inhibitory signals may reduce electrical activity by decreasing Ca^2+^ transients, particularly through the activation of K^+^ currents or direct inhibition of VGCCs [8].

The coupling between Ca^2+^ and secretion is further specialized by Ca^2+^-dependent sensors such as synaptotagmin and calmodulin, which coordinate the fusion of secretory vesicles with the plasma membrane. The coexistence of vesicle populations with differing Ca^2+^ sensitivities enables fine-tuned modulation of release according to physiological context and firing frequency [7,8,67]. Collectively, the modulation of calcium-dependent excitability and exocytosis, the integration of intracellular Ca^2+^ stores, and hormone-mediated feedback mechanisms represent the principal shared pathways underlying neuroendocrine integration.

Notably, these mechanisms are developmentally regulated. In the immature brain and endocrine system, calcium signaling displays heightened plasticity, lower activation thresholds, and prolonged intracellular transients compared with adulthood. As a result, genetic or metabolic perturbations of calcium homeostasis in early life may disrupt neuroendocrine maturation, whereas similar alterations arising in adulthood often produce milder or more organ-restricted phenotypes. Within this developmental context, key calcium signaling components—such as CaSR, VGCCs, and SOCE—act as convergent molecular nodes linking endocrine regulation and neurodevelopment. Although individual disorders differ in genetic etiology or biochemical presentation, disruption of these shared signaling hubs leads to overlapping neuroendocrine phenotypes, supporting calcium signaling as a unifying pathophysiological axis in pediatric disease.

## 3. Genetic Disorders of Calcium Sensing and Signaling

Genetic disorders affecting calcium sensing and signaling primarily involve mutations in *CASR* or in genes encoding proteins that participate in its downstream signaling pathways, including the G-protein α-11 subunit and the adaptor-related protein complex 2 sigma (AP2σ) subunit [68]. Alterations in these components disrupt the ability of cells to appropriately detect and respond to changes in extracellular Ca^2+^, leading to characteristic disturbances in calcium homeostasis.

Additional genetic defects involve store-operated calcium entry (SOCE) machinery. CRAC channels are composed of ORAI1 proteins localized in the plasma membrane and are activated by STIM1 and STIM2, which function as ER Ca^2+^ sensors [69]. Mutations in ORAI1 or STIM genes impair SOCE, resulting in combined immunodeficiency, muscle weakness, and neurodevelopmental abnormalities due to defective Ca^2+^ signaling across multiple systems.

Other genetic alterations affect VGCCs, including CACNA1A and CACNA1C. Pathogenic variants in these channels are associated with neurological and neurodevelopmental disorders such as epilepsy, familial hemiplegic migraine, autism spectrum disorder, and Timothy syndrome, reflecting the essential role of Ca^2+^ influx in neuronal excitability, synaptic transmission, and brain development [70,71].

### 3.1. CASR-Related Disorders

Mutations in *CASR* gene can be broadly classified into two functional categories: loss-of-function and gain-of-function. Loss-of-function mutations are responsible for a spectrum of hypercalcemic disorders, including NSHPT and FHH, whereas gain-of-function mutations lead to ADH1, which may present with a Bartter syndrome-like phenotype [72].

NSHPT (OMIM 239200) is a rare and severe genetic disease, with approximately 100 cases reported to date [73]. It is most often caused by homozygous or compound heterozygous inactivating mutations in the *CASR* gene, resulting in near-complete loss of receptor function. The occurrence of both NSHPT and FHH within the same families, together with frequent parental consanguinity in NSHPT, supports the concept that NSHPT arises from homozygous variants, whereas heterozygous variants cause the milder FHH phenotype [72]. NSHPT typically presents within the first six months of life, often in the early postnatal period, with severe PTH-dependent hypercalcemia (serum calcium > 4 mmol/L), parathyroid gland hyperplasia, skeletal demineralization, and failure to thrive. Additional findings may include polyuria, dehydration, and hypotonia [74]. If untreated, NSHPT can be fatal or lead to serious complications during infancy [73]. The gold standard treatment is total parathyroidectomy with complete removal of all parathyroid tissue, using intraoperative PTH monitoring to confirm surgical success [75]. Parathyroid autotransplantation may be attempted, although long-term endocrine function is frequently inadequate. Some patients improve without surgery through intensive medical management, including intravenous hydration and loop diuretics to enhance renal calcium excretion, bisphosphonates, calcimimetics, dialysis, or respiratory support [73].

FHH is a generally benign disorder inherited in an autosomal dominant pattern. Three genetic subtypes have been identified: FHH1, FHH2, and FHH3. FHH1 (OMIM #145980), the most common form, is caused by heterozygous inactivating mutations in *CASR* on chromosome 3 [76]. It is characterized by lifelong, nonprogressive mild to moderate hypercalcemia, relative hypocalciuria (calcium/creatinine clearance ratio < 0.01), and PTH levels that are normal or slightly elevated. Mild hypermagnesemia may also be observed. Symptoms are usually minimal, although some individuals report fatigue, weakness, or cognitive difficulties; rare complications include pancreatitis and chondrocalcinosis [77,78]. FHH2 (OMIM #145981) results from heterozygous loss-of-function mutations in GNA11 on chromosome 19p13.3, which encodes the G-protein α-11. Its phenotype closely resembles FHH1. FHH3 (OMIM #600740) is caused by heterozygous loss-of-function mutations in *AP2S1* on chromosome 19q13.3, encoding the AP2σ subunit [79]. FHH3 typically exhibits more pronounced biochemical abnormalities than FHH1 and FHH2, including higher serum calcium and magnesium levels and more markedly reduced renal calcium excretion. Cognitive and behavioral disturbances in children have also been reported in association with FHH3 [68,80].

ADH1 is a hereditary disorder characterized by low serum calcium due to inappropriate suppression of PTH secretion. ADH1 is the most common genetic cause of isolated hypoparathyroidism and results from gain-of-function mutations in *CASR*, which increase receptor sensitivity to extracellular calcium and suppress PTH release even at normal Ca^2+^ concentrations [81]. The biochemical profile includes mild to moderate hypocalcemia, elevated phosphate, increased urinary calcium excretion, and low but detectable PTH levels. Clinical manifestations depend on the degree of hypocalcemia and range from neuromuscular irritability to cramps, paresthesias, tetany, and—in more severe cases—seizures. Hyperphosphatemia, hypomagnesemia, nephrocalcinosis, nephrolithiasis, and occasional basal ganglia calcifications may occur [82]. Autosomal dominant hypocalcemia type 2 is caused by activating mutations in GNA11 and produces biochemical and clinical features that largely mirror ADH1 [81]. Diagnosis is based on clinical evaluation, characteristic laboratory findings, and genetic testing for *CASR* or *GNA11* variants [83].

### 3.2. STIM1 and ORAI1 Defects

Mutations in STIM1 and ORAI1 disrupt the SOCE pathway, a fundamental mechanism for maintaining intracellular Ca^2+^ homeostasis. STIM1 is located in the ER, where it functions as a calcium sensor, while ORAI1 forms a highly calcium-selective ion channel in the plasma membrane. Under physiological conditions, depletion of ER calcium stores activates STIM1, which then translocates toward ER–plasma membrane junctions to interact with ORAI1. This interaction opens the CRAC channel, allowing extracellular Ca^2+^ influx to restore ER calcium levels and sustain intracellular calcium signaling [84].

Loss-of-function mutations in STIM1 or ORAI1 impair SOCE and lead to defective calcium signaling in multiple tissues, particularly lymphocytes and skeletal muscle cells [69]. Clinically, these mutations present with a syndrome resembling severe combined immunodeficiency, characterized by impaired T-cell activation and cytokine production, despite largely preserved lymphocyte development. Patients experience recurrent severe bacterial and viral infections and immune dysregulation, including autoimmune hemolytic anemia and immune thrombocytopenia, especially in STIM1 deficiency. Lymphoproliferation may also occur. Non-immunologic features include nonprogressive muscle hypotonia due to impaired calcium-dependent contraction, as well as ectodermal dysplasia manifested by anhidrosis and dental enamel defects [85]. Additional abnormalities may include reduced regulatory T cells and invariant natural killer T cells, contributing to immune dysregulation and autoimmunity. Some patients show elastic skin, joint hyperlaxity, hypoplastic patellae, and dysmorphic facial features, underscoring the broad developmental importance of SOCE [86].

Gain-of-function mutations in STIM1 and ORAI1 have also been described. These variants, typically inherited in an autosomal dominant manner, cause constitutive CRAC channel activation and excessive Ca^2+^ influx through the SOCE pathway. Clinically, they are primarily associated with tubular aggregate myopathy and Stormorken syndrome, which feature muscle weakness, cramps, miosis, thrombocytopenia, hyposplenism, ichthyosis, and short stature, reflecting widespread dysregulation of intracellular calcium homeostasis [69].

### 3.3. TRPV6-Related Diseases

TRPV6 is a highly calcium-selective epithelial ion channel of the TRP family, expressed predominantly in the intestine, kidney, placenta, epididymis, and several exocrine tissues. It mediates apical calcium entry into epithelial cells and represents a key regulator of transcellular calcium absorption, especially in the intestine and placenta. TRPV6 activity supports vitamin D-dependent intestinal calcium uptake and becomes particularly important under conditions of low dietary calcium intake [51].

Alterations in TRPV6 function have been implicated in several human diseases. Biallelic loss-of-function mutations impair placental calcium transport, leading to transient neonatal hyperparathyroidism, a condition characterized by elevated PTH, skeletal demineralization, and radiographic bone abnormalities that typically resolve after birth once normal neonatal calcium homeostasis is established [52]. Mutations in the *TRPV6* gene have also been associated with early-onset chronic pancreatitis, particularly in non-alcoholic individuals, suggesting a role for dysregulated epithelial calcium handling in pancreatic injury and inflammation [87].

Conversely, overexpression or dysregulation of TRPV6 contributes to the pathogenesis and progression of several epithelial malignancies, including prostate, breast, ovarian, and pancreatic cancers. Elevated TRPV6 expression correlates with tumor aggressiveness, metastatic behavior, and poor clinical outcomes, and the channel is under investigation as a potential therapeutic target in oncology [52].

Neurological involvement in TRPV6-related disease remains poorly defined in humans; however, experimental studies provide supportive evidence for a potential role. Altered TRPV6 expression influences axonal growth and synaptic circuit formation, particularly in the hippocampus, and mouse models have suggested possible contributions to neuronal plasticity and seizure susceptibility [88].

## 4. Metabolic and Acquired Disorders with Calcium Signaling Dysfunction

Metabolic and acquired disorders affecting calcium signaling primarily arise from alterations in calcium regulation due to genetic mutations, receptor dysfunction, or systemic conditions that disrupt intracellular signaling and calcium homeostasis. Among the acquired disorders, anti-CaSR autoantibodies represent a notable mechanism capable of inducing either hypocalcemia or hypercalcemia, as observed in autoimmune polyendocrinopathy type 1 (APS-1) and in certain forms of autoimmune hypoparathyroidism [89,90]. Additional acquired causes include primary hyperparathyroidism, vitamin D deficiency, chronic kidney disease, and malignancy-associated overproduction of PTH-related peptide [21,24,27].

These conditions can alter calcium signaling at the cellular level, leading to abnormalities in bone remodeling, neuromuscular excitability, hormonal secretion, and neuronal function [91,92]. Diagnostic evaluation requires biochemical assessment of calcium, phosphate, PTH, vitamin D, and magnesium levels, complemented by genetic testing when hereditary forms are suspected. In acquired disorders, the detection of autoantibodies and the evaluation of underlying systemic or endocrine diseases are essential for accurate diagnosis and management.

### 4.1. Primary and Secondary Hypoparathyroidism

Primary and secondary hypoparathyroidism typically present with hypocalcemia, which leads to neuromuscular irritability, including paresthesias and muscle cramps, as well as tetany and, in severe cases, seizures and potentially life-threatening complications such as laryngospasm and cardiac arrhythmias. Chronic hypocalcemia may cause neuropsychiatric manifestations and prolongation of the QT interval. Intracranial calcifications—especially within the basal ganglia—and other forms of ectopic calcification are common findings, largely attributable to hyperphosphatemia resulting from the loss of PTH activity at the renal level [93,94,95]. In children, hypoparathyroidism is frequently of genetic origin, with 22q11.2 deletion syndrome representing the most common cause.

Other etiologies include mutations in *AIRE* associated with APS-1, mutations in *GATA3*, and activating mutations of *CASR*. In the pediatric population, seizures are often the earliest presenting symptom, typically occurring in the neonatal period or early infancy. Additional clinical features include muscle cramps, tremors, rigidity, irritability, and QT prolongation. Cerebral calcifications and renal complications—such as nephrocalcinosis and nephrolithiasis—are observed in a substantial proportion of affected children.

Outcomes vary considerably. Hypoparathyroidism may be transient, particularly in children with 22q11.2 deletion syndrome, or permanent. Transient forms may resolve with growth or following correction of precipitating factors. Permanent hypoparathyroidism requires long-term treatment with calcium supplementation and active vitamin D analogs such as calcitriol. The American Academy of Pediatrics recommends regular monitoring of serum calcium, phosphate, and renal function to minimize complications and optimize long-term outcomes [25].

### 4.2. Pseudohypoparathyroidism and GNAS-Related Disorders

Pseudohypoparathyroidism (PHP) refers to a group of rare genetic and epigenetic disorders characterized by resistance to PTH at target organs, resulting in hypocalcemia, hyperphosphatemia, and elevated PTH despite normal renal function. The underlying defect typically involves loss of function of the stimulatory G-protein alpha subunit, encoded by the imprinted *GNAS* locus on chromosome 20q13.3. The clinical spectrum includes several subtypes, most notably PHP type 1A (PHP1A), PHP type 1B (PHP1B), and pseudopseudohypoparathyroidism (PPHP), collectively referred to as *GNAS*-related disorders [96,97,98,99,100,101,102].

PHP1A is caused by maternally inherited inactivating mutations in exons 1–13 of *GNAS*. These mutations lead to end-organ PTH resistance and to features of Albright hereditary osteodystrophy (AHO), including short stature, brachydactyly, subcutaneous ossifications, and, in some patients, cognitive impairment. In contrast, the same mutations inherited from the father result in PPHP, which presents the AHO phenotype without hormonal resistance [96,97,98,99,100,101,103,104].

PHP1B is most commonly associated with epigenetic abnormalities at the *GNAS* locus, particularly loss of methylation at exon A/B, and is characterized by isolated PTH resistance with or without mild AHO-like features [96,98,99,101,105,106].

Other disorders within the *GNAS* spectrum include progressive osseous heteroplasia, a severe condition marked by heterotopic ossification and typically associated with paternally inherited *GNAS* mutations [100,101,104]. Phenotypic variability across *GNAS*-related disorders reflects the parental origin of the mutation due to tissue-specific imprinting of *GNAS*, which determines the extent of stimulatory G-protein alpha subunit expression in selected tissues such as the renal proximal tubule [97,98,99,104,106].

Diagnosis relies on a combination of clinical findings, biochemical evaluation, and genetic and epigenetic testing. Management is supportive and focuses on correcting hypocalcemia and hyperphosphatemia, typically through oral calcium supplementation and active vitamin D analogues. At present, no therapies are available that modify the natural course of the disease [101,102].

Alterations in PTH signaling are associated with cognitive deficits and characteristic behavioral phenotypes. In patients with hypoparathyroidism, neuropsychological disturbances are common and include reduced inhibitory control, visuospatial deficits, psychomotor slowing, and impaired executive function. These manifestations reflect low serum calcium levels as a direct consequence of impaired PTH signaling and may be partially reversible with restoration of calcium homeostasis [107,108,109,110].

In PHP1A, which involves resistance to PTH and additional hormones due to *GNAS* mutations, cognitive impairment is prominent and includes reduced intelligence quotient, executive dysfunction, delayed behavioral adaptation, and an increased prevalence of ADHD. Approximately 80% of individuals with PHP1A exhibit cognitive impairment, whereas patients with PPHP—who share the skeletal phenotype but do not exhibit hormonal resistance—generally do not show such deficits. This contrast supports a mechanistic link between PTH resistance and neurocognitive dysfunction [111,112,113]. Animal models of PHP1A reinforce this concept, showing impaired amygdala-dependent learning, decreased anxiety, and increased impulsivity, consistent with the behavioral phenotypes observed in affected individuals [8].

In summary, impaired PTH signaling contributes to cognitive deficits and behavioral changes, with severity influenced by the underlying etiology and by the degree of calcium dysregulation.

### 4.3. Vitamin D-Related Disorders

Vitamin D-related disorders encompass a broad spectrum of conditions arising from abnormalities in vitamin D metabolism, action, or nutritional status. The most common disorders are vitamin D deficiency and insufficiency, which classically manifest as rickets in children and osteomalacia in adults due to impaired bone mineralization. These conditions are characterized by hypocalcemia, secondary hyperparathyroidism, and hypophosphatemia, leading to bone pain, muscle weakness, and increased fracture risk. The Endocrine Society emphasizes the causal role of vitamin D deficiency in these musculoskeletal complications and recommends supplementation for both prevention and treatment [114,115,116,117]. Observational studies have also associated low vitamin D levels with increased risk of metabolic, cardiovascular, autoimmune, infectious, and neoplastic diseases; however, causality remains unproven, and the benefits of supplementation outside bone health continue to be uncertain [117,118,119,120,121].

Vitamin D-dependent rickets (VDDR) includes hereditary disorders affecting vitamin D metabolism or receptor function, such as VDDR type 1A (1α-hydroxylase deficiency), type 1B (25-hydroxylase deficiency), and type 2A (vitamin D receptor mutations). These disorders present with early-onset rickets, hypocalcemia, and, in type 2A, frequently alopecia [114]. The most significant neurological complications associated with rickets and hypocalcemia include hypocalcemic seizures, tetany, carpopedal spasm, neuromuscular irritability, and, in rare cases, developmental regression. Hypocalcemic seizures represent a well-documented and potentially life-threatening manifestation, particularly in infants and young children with nutritional rickets due to vitamin D deficiency or inadequate calcium intake [122,123,124,125,126]. Tetany—characterized by involuntary muscle contractions and spasms, including carpopedal spasm—is another classic sign of hypocalcemia [122,127]. Additional neurological features include hypotonia and delayed motor milestones, reflecting impaired neuromuscular function secondary to chronic calcium deficiency. Rarely, affected children may experience developmental regression, which can improve after restoration of calcium and vitamin D levels [122,128]. Other symptoms, such as irritability, jitteriness, and, in severe cases, laryngospasm or stridor, may result from increased neuromuscular excitability [122,129,130].

The underlying pathophysiology relates to low serum calcium, which increases neuronal excitability and lowers the threshold for action potential generation, thereby predisposing to the observed neurological manifestations. Prompt identification and correction of hypocalcemia are crucial to prevent permanent neurological sequelae. Treatment is tailored to the specific etiology: nutritional deficiency is treated with vitamin D supplementation (typically 400–800 IU per day for prevention), whereas hereditary forms often require active vitamin D analogues or other targeted therapies [114,117].

Vitamin D-related hypercalcemia may occur due to excessive vitamin D intake, increased endogenous production—as seen in granulomatous diseases—or genetic defects in vitamin D degradation. This condition can lead to hypercalcemia, hypercalciuria, and nephrocalcinosis [131].

### 4.4. Acquired Causes

Acquired causes of altered calcium homeostasis—which secondarily disrupt calcium-dependent signaling—include post-surgical hypoparathyroidism, autoimmune hypoparathyroidism, and neonatal hypocalcemia.

Post-surgical hypoparathyroidism most commonly results from inadvertent removal or irreversible damage to the parathyroid glands during anterior neck surgeries, particularly thyroidectomy or parathyroidectomy. It is characterized by hypocalcemia with low or inappropriately normal PTH levels and typically appears within days to weeks after surgery. Permanent hypoparathyroidism is defined by persistent hypocalcemia and low PTH lasting more than six months postoperatively. This etiology accounts for the majority of adult cases and is associated with an increased risk of renal complications and soft tissue calcifications due to long-term treatment with calcium and active vitamin D supplementation [93,95,132,133,134].

Autoimmune hypoparathyroidism results from immune-mediated destruction of the parathyroid glands. It may occur as an isolated condition or as part of APS-1, which is characterized by hypoparathyroidism, chronic mucocutaneous candidiasis, and adrenal insufficiency. Diagnosis is suggested by the coexistence of other autoimmune diseases and may be supported by characteristic clinical findings. Unlike post-surgical hypoparathyroidism, autoimmune forms can present at any age and are frequently associated with additional endocrinopathies [132,135,136].

Neonatal hypocalcemia is distinct from hypoparathyroidism and refers to low serum calcium levels in newborns, typically occurring within the first days of life. It is most often caused by transient conditions such as prematurity, perinatal asphyxia, maternal diabetes, or vitamin D deficiency, rather than intrinsic parathyroid dysfunction. Rarely, neonatal hypocalcemia is due to congenital hypoparathyroidism, which may be genetic or associated with syndromes such as 22q11.2 deletion syndrome; these cases are not related to autoimmune or post-surgical etiologies [93,132,136].

## 5. Diagnostic Challenges in Pediatric Calcium Signaling Disorders

### 5.1. Biochemical and Hormonal Assays

The concentrations of PTH, vitamin D, calcium, phosphate, and magnesium fluctuate throughout childhood and adolescence and are influenced by age, sex, and season [137,138]. In the evaluation of calcium metabolism in pediatric patients, it is essential to assess both total calcium (tCa) and ionized calcium (iCa). Total calcium comprises the ionized, biologically active fraction as well as the portion bound to plasma proteins (primarily albumin) and anions. However, only ionized calcium reflects the fraction truly available for cellular physiological processes [139]. In clinical practice, tCa does not always correspond to iCa, and a significant proportion of patients, including those with histologically confirmed parathyroid disease, may present with isolated ionized hypercalcemia at diagnosis.

Theoretically, iCa can be estimated from tCa, but this approach is unreliable under various biological conditions, such as abnormal albumin or globulin levels, critical illness, chronic liver or kidney disease, or following liver transplantation. Artificially altered tCa values may also result from the presence of anticoagulants or calcium-chelating agents. Interindividual differences in calcium–albumin binding further reduce the accuracy of correction formulas based on population averages. Consequently, direct measurement of iCa remains the most reliable indicator of calcium homeostasis [140], although it is important to recognize that iCa levels fluctuate markedly in the first week of life and gradually stabilize to reach adult-like concentrations as children grow [141].

PTH levels in pediatric patients must be interpreted using age- and sex-specific reference ranges, as PTH concentrations vary considerably throughout childhood and adolescence and are also influenced by season and vitamin D status. For example, vitamin D deficiency, which is very common in children, can induce secondary hyperparathyroidism, leading to elevated PTH in the absence of a primary parathyroid disorder [132]. Similarly, chronic kidney disease, hypomagnesemia, and other comorbid conditions can cause secondary alterations in PTH, often with increased levels driven by compensatory mechanisms rather than by primary calcium signaling or intrinsic parathyroid pathology [138].

Assessment of vitamin D status is a key component of the diagnostic work-up in children with calcium disorders. Serum total 25-hydroxyvitamin D (25(OH)D) is the most commonly used marker, but no pediatric clinical indicator with sufficient sensitivity has been clearly identified. Levels of 25(OH)D may be misleading due to the influence of vitamin D binding protein (DBP), which affects total 25(OH)D without necessarily reflecting the biologically active fraction, particularly in neonates, where DBP differs from that of older children [142]. Moreover, the relationship between 25(OH)D and PTH is inconsistent in pediatric populations: newborns have lower PTH thresholds for vitamin D deficiency, and guideline cut-offs show considerable variability [143].

Serum phosphate and magnesium are also highly age dependent and influenced by developmental, genetic, and nutritional factors. Phosphate concentrations are naturally higher in neonates and infants and decline with age; thus, age-adjusted reference intervals are essential to avoid misdiagnosis [144]. Interpretation of magnesium is similarly complex, as levels are affected by gestational age, renal function, and nutritional status, and hypomagnesemia can exacerbate hypocalcemia by impairing PTH secretion [145,146]. Rare genetic tubulopathies add further complexity, while assay variability and the lack of standardized pediatric protocols introduce additional challenges in interpretation [145].

### 5.2. Genetic Testing and Molecular Diagnostics

Genetic testing, including targeted gene panels and whole-exome or whole-genome sequencing, is crucial for diagnosing pediatric calcium-sensing and signaling disorders, particularly when clinical or biochemical findings are unclear or suggest a monogenic etiology [Table 1]. Targeted panels can efficiently detect known pathogenic variants in genes such as *CASR*, *GNA11*, *AP2S1*, and *CYP24A1*, but may miss rare or novel mutations [147]. Exome sequencing provides broader coverage of coding regions and increases diagnostic yield, while whole-genome sequencing can identify deep intronic, regulatory, structural, and small copy-number variants that might be overlooked by exome-based approaches [148].

In critically ill neonates and infants, rapid exome or genome sequencing can deliver timely and actionable diagnoses that guide management. These methods can, for example, distinguish FHH from primary hyperparathyroidism, thereby preventing unnecessary surgery, or identify severe neonatal hyperparathyroidism caused by biallelic *CASR* mutations, which may require urgent intervention [149]. Genotype–phenotype correlations, such as the greater severity associated with homozygous *CASR* variants, inform prognosis and therapeutic decisions. Identification of pathogenic variants also facilitates cascade testing and genetic counseling for family members [73].

The American Academy of Pediatrics recommends exome or genome sequencing as a first-line test for suspected genetic disorders, highlighting their high diagnostic yield and potential cost-effectiveness, while emphasizing the importance of careful variant interpretation and the need for periodic re-evaluation as knowledge and databases evolve [150].

**Table 1:** Genetic determinants of calcium sensing and signaling disorders: gene function, mutation type, and associated phenotypes.

Gene/Factor	Gene Function/Role	Type of Mutation/Alteration	Associated Disorder
CASR	Calcium-sensing receptor; regulates PTH secretion	Loss-of-function	Familial hypocalciuric hypercalcemia 1, neonatal severe hyperparathyroidism [72,76]
		Gain-of-function	Autosomal dominant hypocalcemia type 1—autosomal dominant familial hypoparathyroidism [72]
GNA11	Gα11 subunit of G protein; transduces the CaSR signal	Loss-of-function	Familial hypocalciuric hypercalcemia 2 [79]
		Gain-of-function	Autosomal dominant hypocalcemia type 2—autosomal dominant familial hypoparathyroidism type 2 [81]
AP2S1	σ subunit of adaptor protein 2 (involved in CaSR trafficking)	Loss-of-function	Familial hypocalciuric hypercalcemia 3—familial hypercalcemia with reduced renal calcium excretion [79]
STIM1	Endoplasmic reticulum calcium sensor; activates calcium release-activated calcium channel	Loss-of-function	Severe combined immunodeficiency-like immunodeficiency, muscle hypotonia, ectodermal dysplasia [69,86]
		Gain-of-function	Stormorken syndrome, tubular aggregate myopathy [69]
ORAI1	Calcium-selective ion channel in the plasma membrane	Loss-of-function	Severe combined immunodeficiency-like immunodeficiency, muscle hypotonia, ectodermal dysplasia [69,86]
		Gain-of-function	Stormorken syndrome, tubular aggregate myopathy [69]
TRPV6	Highly calcium-selective epithelial channel; intestine, placenta, kidney	Biallelic loss-of-function	Transient neonatal hyperparathyroidism, skeletal demineralization [52]
CACNA1A, CACNA1C	Voltage-gated calcium channels	Different mutation types	Epilepsy, familial hemiplegic migraine, Timothy syndrome, autism [70,71]
GNAS	Gsα (stimulatory G protein subunit); mediates the PTH signal	Maternal loss-of-function	Pseudohypoparathyroidism type 1A—PTH resistance + AHO [96,97,98,99,100,101,103,104]
		Paternal loss-of-function	Pseudopseudohypoparathyroidism—skeletal AHO phenotype without hormonal resistance [96,97,98,99,100,101,103,104]
		Epigenetic defects (loss of methylation)	Pseudohypoparathyroidism type 1B—isolated PTH resistance, ±mild AHO features [96,98,99,101,105,106]
		Paternal loss-of-function	Progressive heterotopic ossification [100,101,104]
AIRE	Regulates immune tolerance; prevents autoimmunity	Loss-of-function	Autoimmune polyendocrinopathy type 1—autoimmune hypoparathyroidism [151]
GATA3	Transcription factor; parathyroid and auditory system development	Loss-of-function	HDR syndrome (hypoparathyroidism, deafness, renal dysplasia) [152]
CYP27B1 (1α-hydroxylase)	Converts 25(OH)D (25-hydroxyvitamin D) into active 1,25(OH)_2_D (calcitriol)	Loss-of-function	Vitamin D-dependent rickets type 1A—rickets, hypocalcemia [114]
CYP2R1 (25-hydroxylase)	Vitamin D hydroxylation in the liver	Loss-of-function	Vitamin D-dependent rickets type 1B—rickets, hypocalcemia [114]
VDR (vitamin D receptor)	Mediates vitamin D effects on bone and calcium	Loss-of-function	Vitamin D-dependent rickets type 2A—rickets, hypocalcemia, alopecia [114]

Abbreviations: AHO, Albright hereditary osteodystrophy; CaSR, calcium-sensing receptor; PTH, parathyroid hormone.

### 5.3. Neuroimaging and Functional Studies

Neuroimaging and functional studies play a crucial role in the diagnosis and management of calcium-sensing and signaling disorders. Basal ganglia calcifications are the most common neuroradiological findings in these patients. Computed tomography is the gold standard for detecting and quantifying these calcifications, which may also involve the thalamus, cerebellum, and subcortical white matter [153]. Although the extent and distribution of calcifications often correlate with clinical severity, they do not consistently predict neurological symptoms, and some individuals remain asymptomatic despite widespread deposits [154].

Functional imaging techniques, such as fluorodeoxyglucose positron emission tomography and single-photon emission computed tomography, in symptomatic patients have demonstrated regional hypometabolism and reduced perfusion, particularly in the striatum and cortical regions such as the precuneus and posterior cingulate, which are involved in memory and executive functions. These findings suggest that neurological deficits arise more from disruption of neuronal networks than from calcifications per se [155].

In children with disorders involving *CASR*, *STIM1* and *ORAI1* defects, or TRPV6-related conditions, the electroencephalogram frequently shows diffuse background slowing with pleomorphic focal or multifocal epileptiform discharges. These patterns are common in genetic epilepsies of infancy and are associated with significant developmental delay and persistent seizures. Although such electroencephalogram findings are not specific to calcium disorders, their presence—particularly in combination with developmental delay and normal structural neuroimaging—should raise suspicion for a calcium channelopathy and prompt consideration of genetic testing [156].

From a neuropsychological perspective, children with calcium-sensing and signaling disorders display a wide range of impairments, including global developmental delay, intellectual disability, motor and language deficits, learning difficulties, and neuropsychiatric symptoms such as autism, ADHD, and epilepsy [157,158]. Calcium signaling also modulates neurotrophin pathways, crucial for synaptic plasticity and neurodevelopment; altered Ca^2+^ dynamics may therefore contribute to cognitive and behavioral phenotypes in pediatric neuroendocrine disorders [159]. The severity and specific profile vary according to gene and mutation type. Activating *CASR* mutations can lead to seizures and cognitive deficits, sometimes exacerbated by basal ganglia calcifications [160]. Inactivating *CASR* mutations rarely affect cognition unless hypercalcemia is severe [72]. Loss-of-function mutations in *STIM1* and *ORAI1* are associated with developmental delay, intellectual disability, and hypotonia, whereas gain-of-function mutations tend to cause milder cognitive or learning difficulties [69]. TRPV6-related disorders have not shown a consistent neuropsychological impact in humans [161]. Despite this heterogeneity, these conditions consistently impact development, cognition, motor function, and behavior, underscoring the central role of calcium signaling in neurodevelopment [162].

### 5.4. Diagnostic Pitfalls and Overlapping Phenotypes

Calcium-sensing and signaling disorders are frequently misdiagnosed because their manifestations—such as seizures, movement disorders, cognitive impairment, and psychiatric symptoms—closely resemble those of primary neurological or psychiatric conditions [163]. For example, ADH1 may present with neuromuscular irritability, seizures, and neuropsychiatric symptoms, yet it is often overlooked or misclassified as a primary neurological disorder [82]. Similarly, FHH and other CaSR-related conditions may present with subtle or nonspecific neurological signs, further complicating the diagnostic process.

Accurate diagnosis requires integration of biochemical, genetic, and imaging data with careful clinical evaluation. Measurement of serum calcium, phosphate, magnesium, and PTH levels is essential to identify underlying disturbances in calcium homeostasis. Genetic testing can confirm pathogenic variants in key genes, clarifying the disease mechanism. Neuroimaging that reveals characteristic brain calcifications may indicate an organic etiology rather than a primary neurodegenerative process. Finally, a positive family history or the presence of systemic features—such as renal, cardiac, or endocrine involvement—should prompt consideration of an underlying calcium-sensing or signaling disorder.

## 6. Therapeutic Strategies and Emerging Approaches

Therapeutic strategies for pediatric calcium signaling disorders can be broadly divided into established standard-of-care interventions aimed at correcting biochemical abnormalities and symptom control, and emerging precision approaches targeting the underlying molecular defects.

### 6.1. Conventional Treatments

Conventionally established management of calcium-sensing and calcium-signaling disorders focuses on correcting disturbances in calcium balance and PTH regulation. In hypocalcemic conditions, such as ADH1, treatment typically involves oral calcium and active vitamin D analogues (e.g., calcitriol). The therapeutic aim is to maintain serum calcium levels at the lower end of the normal range while minimizing symptoms. However, this approach frequently leads to hypercalciuria, increasing the risk of nephrocalcinosis and, over time, renal impairment. PTH replacement therapy (e.g., recombinant human PTH 1–34) can reduce urinary calcium excretion compared with standard therapy, although it does not fully prevent complications such as soft-tissue calcifications [164,165]. Dietary strategies, including moderate calcium intake and adequate hydration, are often recommended to further reduce renal risk.

In hypercalcemic disorders such as FHH, most individuals remain asymptomatic and do not require active treatment. When symptoms occur, management focuses on hydration and, in rare cases, pharmacologic intervention [139].

### 6.2. Precision Targeting Calcium Signaling: Calcilytics and Calcimimetics

Calcimimetics and calcilytics represent prototypical examples of precision medicine in inherited calcium-sensing disorders, as their therapeutic use is directly guided by the underlying molecular defect and the direction of CaSR signaling dysfunction. These allosteric modulators of the CaSR have potential therapeutic applications particularly in disorders caused by mutations in *CASR*, *GNA11*, and *AP2S1*, and, to a lesser extent, *STIM1* [166].

Calcimimetics (e.g., cinacalcet) are positive allosteric modulators that enhance CaSR activity and are used to treat hypercalcemic conditions such as FHH and NSHPT. They lower serum calcium and PTH levels in symptomatic patients and may reduce the need for parathyroid surgery [167].

Calcilytics (e.g., NPS 2143, JTT-305/MK-5442, AXT914) are negative allosteric modulators that inhibit CaSR activity and are under investigation for the treatment of ADH1. In preclinical models, they normalize serum calcium, increase endogenous PTH secretion, and reduce hypercalciuria and renal calcifications [164]. Quinazolinone calcilytics (AXT914, ATF936) have demonstrated mutation-specific efficacy in mouse models of ADH1 [168].

In disorders involving *GNA11* and *AP2S1*, both calcimimetics and calcilytics can correct signaling and trafficking defects in cellular models, supporting the potential for mutation-specific targeted therapy [169]. Evidence for STIM1-related disorders remains limited, as STIM1 is not directly part of the CaSR signaling pathway [170].

### 6.3. Experimental Gene-Based Precision Strategies

Gene therapy and molecular approaches such as CRISPR/Cas9 genome editing or antisense oligonucleotides represent emerging forms of gene-based precision medicine for calcium-sensing and signaling disorders. However, these modalities remain experimental and are not yet available for clinical use. Future advances in vector technology, delivery systems, and safety optimization may eventually allow targeted correction of pathogenic variants in disorders involving *CASR*, *GNA11*, *AP2S1*, or SOCE-related genes.

Despite their promise, several critical limitations currently hinder the clinical translation of gene-based therapies in calcium signaling disorders. Major challenges include efficient and tissue-specific delivery to endocrine glands and the central nervous system, limited targeting of post-mitotic cells, and the risk of off-target genomic alterations, particularly with genome-editing approaches. In addition, long-term safety data are lacking, especially in pediatric populations, where permanent genomic modifications raise ethical and developmental concerns. For antisense-based strategies, issues related to sustained efficacy, repeated administration, and systemic distribution remain unresolved. Progress toward clinical application will require the development of safer delivery vectors, improved control of on-target specificity, robust long-term follow-up data, and disease-specific outcome measures capable of capturing both endocrine and neurodevelopmental benefits.

### 6.4. Neuroprotective and Multidisciplinary Approaches

Management of developmental and drug-resistant epilepsies associated with calcium signaling disorders requires a multidisciplinary, individualized approach. Seizure control relies on standardized treatment protocols, tailored anti-seizure medication selection, therapeutic drug monitoring, and periodic assessment of both efficacy and side effects. Emerging therapeutic strategies, including sigma-1 receptor modulation, show promise for reducing seizures and improving cognitive outcomes [171].

Neurodevelopmental care should include baseline and follow-up neuropsychological evaluations, personalized behavioral and educational interventions, and physical and occupational therapy as needed [25]. Early intervention and continuous monitoring are essential for optimizing developmental and cognitive outcomes. Long-term follow-up should address seizure control, developmental progress, comorbidities such as contractures or feeding difficulties, behavioral challenges, and family support needs. Effective management often requires coordinated multidisciplinary care, including neurology, endocrinology, rehabilitation services, social work, and access to community resources.

## 7. Clinical and Translational Implications

Despite the apparent heterogeneity of pediatric calcium signaling disorders, many share common neurodevelopmental and neuropsychiatric consequences driven by disruption of a limited number of calcium-dependent signaling pathways [Table 2]. Recognizing these shared mechanisms facilitates a unified diagnostic and translational framework that moves beyond single-disease descriptions.

In pediatric practice, disorders of calcium signaling may be underdiagnosed or misclassified due to nonspecific neurological presentations and fluctuating biochemical findings. Seizures, hypotonia, developmental delay, or behavioral abnormalities are frequently attributed to primary neurological conditions, delaying recognition of an underlying calcium–phosphate disorder. In addition, transient normalization of serum calcium—particularly in early infancy or during intercurrent illness—may obscure the diagnosis of hypoparathyroidism or CaSR-related conditions.

A systematic biochemical evaluation, including serum calcium, phosphate, magnesium, PTH, and vitamin D metabolites, should therefore be incorporated early in the diagnostic workflow of children presenting with unexplained seizures or neuromuscular hyperexcitability. Failure to recognize inappropriate PTH responses or subtle hypocalciuria may lead to misdiagnosis and suboptimal treatment. Awareness of these diagnostic pitfalls is essential to guide timely genetic testing, avoid unnecessary investigations, and ensure appropriate long-term management.

Early diagnosis of calcium–phosphate homeostasis disorders—including primary and secondary hypoparathyroidism, pseudohypoparathyroidism, *GNAS*-related disorders, and conditions associated with vitamin D deficiency—is essential to prevent acute complications and long-term sequelae. The American Academy of Pediatrics recommends regular monitoring of serum calcium and parathyroid function in children with genetic syndromes such as 22q11.2 deletion syndrome, in whom hypoparathyroidism and hypocalcemia may manifest with convulsive crises, tetany, and neuromuscular irritability. Timely diagnosis is critical to avoid potentially life-threatening complications such as laryngospasm, arrhythmias, and severe hypocalcemic episodes [25].

Pediatric calcium signaling disorders differ from their adult counterparts in both presentation and long-term impact. In children, hypocalcemia or hypercalcemia frequently manifests with seizures, neurodevelopmental delay, hypotonia, and behavioral abnormalities, reflecting the critical role of calcium in brain development. In contrast, adult-onset calcium disorders more commonly present with neuromuscular irritability, renal complications, or neuropsychiatric symptoms without major effects on cognitive development. Early-life exposure to altered calcium signaling therefore carries a disproportionate risk of persistent neurodevelopmental sequelae, underscoring the need for age-specific diagnostic thresholds, monitoring strategies, and therapeutic goals.

Many of these disorders have a genetic or epigenetic basis, including mutations in *AIRE*, *GATA3*, activating variants of *CASR*, and imprinting defects at the *GNAS* locus, requiring multidisciplinary evaluation from the time of diagnosis [26,93,97,136]. Coordinated management among pediatric endocrinologists, neurologists, nephrologists, cardiologists, and geneticists is essential to address the often coexisting endocrine, neurocognitive, renal, and cardiac manifestations. Neuroendocrine monitoring is central to long-term care. Several neuroendocrine hormones involved in social behavior, stress regulation, and emotional processing are secreted through calcium-dependent mechanisms. Altered intracellular calcium signaling may therefore contribute to behavioral and psychosocial vulnerabilities in pediatric endocrine disorders, reinforcing the need for integrated neuroendocrine and neuropsychological follow-up [172]. Chronic hypocalcemia and altered PTH signaling are associated with neurocognitive deficits, including impairments in executive function, visuospatial skills, and attention, along with behavioral phenotypes such as impulsivity and ADHD-like traits, particularly prominent in PHP1A [97,113].

In children with VDDR or genetic hypoparathyroidism, developmental regression, hypotonia, and delayed motor milestones may be present and often improve with correction of biochemical abnormalities. Periodic assessment of calcium, phosphate, PTH, renal function, and markers of mineral metabolism is recommended, along with screening for nephrocalcinosis, basal ganglia calcifications, and conduction abnormalities [25,93,173]. Neuropsychological follow-up is especially important in patients with *GNAS*-related disorders or early-onset, chronic hypocalcemia to identify emerging deficits and guide timely interventions.

### 7.1. Neurological Manifestations of Calcium Signaling Disorders: Central and Peripheral Patterns

Neurological manifestations represent a major source of morbidity in pediatric disorders of calcium signaling and may involve both the central and peripheral nervous systems, with overlapping but distinct patterns across conditions.

Central nervous system involvement is particularly prominent in disorders affecting intracellular calcium homeostasis and synaptic signaling. Common manifestations include seizures, neurodevelopmental delay, cognitive impairment, movement disorders, hypotonia, and behavioral abnormalities. These features are frequently observed in congenital hypoparathyroidism, syndromic calcium disorders (e.g., 22q11.2 deletion syndrome), and conditions involving SOCE defects, reflecting the essential role of calcium in neuronal excitability, synaptic plasticity, and brain maturation.

Peripheral nervous system manifestations are more closely related to altered membrane excitability and neuromuscular transmission. Acute hypocalcemia commonly leads to paresthesias, tetany, carpopedal spasm, and neuromuscular irritability, whereas chronic dysregulation may contribute to peripheral neuropathy or muscle weakness. These features are particularly evident in postsurgical or autoimmune hypoparathyroidism and in metabolic forms of hypocalcemia.

Across disorders, shared neurological patterns include seizures in early life, neuromuscular hyperexcitability, and hypotonia, whereas distinctive features depend on the underlying molecular defect. For example, CaSR-related disorders predominantly affect neuronal excitability through altered extracellular calcium sensing, while SOCE-related defects preferentially impair intracellular calcium dynamics, leading to broader neurodevelopmental phenotypes. Recognition of these recurring neurological patterns may facilitate earlier diagnosis, guide targeted investigations, and inform multidisciplinary management strategies.

### 7.2. Neurodevelopmental Outcomes and Biomarkers

From a clinical perspective, systematic neurodevelopmental follow-up is essential in children with calcium-sensing and signaling disorders. Standardized neurodevelopmental and cognitive tools, such as Bayley Scales in infancy and age-appropriate intelligence and executive-function tests in later childhood, can be used to track global development, learning abilities, and behavioral profiles over time.

Neuroimaging provides complementary structural and functional biomarkers. Basal ganglia and other intracranial calcifications on computed tomography, magnetic resonance imaging signal abnormalities, and functional imaging findings such as regional hypometabolism or hypoperfusion in cortico-striatal networks may serve as imaging correlates of disease severity and neurocognitive risk.

Molecular markers of calcium signaling activity include serum calcium, phosphate, magnesium, PTH, and vitamin D metabolites, as well as gene-specific variants in *CASR*, *GNA11*, *AP2S1*, *STIM1*, *ORAI1*, and *GNAS*. In addition, emerging candidates such as bone-derived hormones (e.g., osteocalcin, FGF-23) and pathway-related proteins involved in store-operated calcium entry may provide more refined readouts of neuroendocrine network dysregulation, although their clinical utility in pediatrics remains to be validated.

## 8. Conclusions and Future Perspectives

Disruptions in calcium sensing and signaling produce complex neuroendocrine consequences, including abnormal PTH secretion, altered serum calcium, and downstream effects on bone metabolism, neuromuscular function, and neurodevelopment. Clinically, these conditions may present with hypercalcemia or hypocalcemia, seizures, neurocognitive impairment, skeletal demineralization, or failure to thrive. A summary of key clinical red flags suggestive of calcium signaling disorders is provided in Table 3, with the aim of supporting early recognition and targeted diagnostic evaluation in pediatric neurology and endocrinology.

Early detection of severe hypercalcemia, such as in homozygous *CASR* mutations, is a key clinical indicator guiding timely therapy. Emerging precision therapies targeting calcium pathways, including calcimimetics and calcilytics, offer opportunities to correct receptor dysfunction and improve biochemical stability.

Future pediatric studies should incorporate standardized longitudinal neurodevelopmental assessments, structured neuroradiological evaluation, and molecular profiling of calcium-related pathways to establish robust outcome measures and biomarkers for clinical trials and long-term monitoring.

Translating mechanistic insights into clinical practice will require coordinated multidisciplinary efforts, with calcium signaling as a unifying framework for managing pediatric neuroendocrine disorders.

## Figures and Tables

**Table 2:** Shared calcium signaling nodes and convergent neuroendocrine phenotypes in pediatric disorders.

Signaling Node	Representative Genes/Pathways	Endocrine Consequences	Neurological/Neurodevelopmental Features	Translational Relevance
CaSR signaling	*CASR*, *GNA11*, *AP2S1*	Hypo-/hypercalcemia, altered PTH secretion	Seizures, neuromuscular irritability, cognitive vulnerability	Targeted therapy (calcimimetics/calcilytics)
SOCE	*STIM1*, *ORAI1*	Hypocalcemia, immune-endocrine dysregulation	Developmental delay, epilepsy, hypotonia	Emerging molecular targets
VGCC-mediated signaling	*CACNA1A*, *CACNA1C*	Hormone secretion defects	Epilepsy, autism spectrum disorder traits, migraine	Channel-modulating therapies
PTH–Gsα signaling	*GNAS*	PTH resistance, mineral imbalance	Cognitive impairment, behavioral phenotypes	Genotype-guided management
Vitamin D pathway	*CYP27B1*, *CYP2R1*, *VDR*	Hypocalcemia, rickets	Hypotonia, delayed milestones	Early biochemical correction

Abbreviations: CaSR, calcium-sensing receptor; PTH, parathyroid hormone; SOCE, store-operated calcium entry; VGCC, voltage-gated calcium channels.

**Table 3:** Clinical red flags suggestive of calcium signaling disorders in pediatric neurology and endocrinology.

Clinical Red Flag	Typical Presentation	Suggested Biochemical Clue	Disorders to Consider
Early-onset seizures (neonatal/infantile)	Refractory or hypocalcemia-responsive	Low Ca^2+^, inappropriately low/normal PTH	Hypoparathyroidism, autosomal dominant hypocalcemia type 1, vitamin D-dependent rickets
Tetany, carpopedal spasm	Triggered by illness or stress	Hypocalcemia, hypomagnesemia	Acquired or genetic hypoparathyroidism
Developmental delay + hypocalcemia	Global delay, hypotonia	Chronic low Ca^2+^, elevated phosphate	Pseudohypoparathyroidism, *GNAS*-related disorders
Hypercalcemia with low urinary Ca^2+^	Often asymptomatic	Elevated Ca^2+^, hypocalciuria	Familial hypocalciuric hypercalcemia (*CASR*, *GNA11*, *AP2S1*)
Neuropsychiatric features (attention deficit hyperactivity disorder-like, autism spectrum disorder traits)	Executive dysfunction, impulsivity	Chronic Ca^2+^/PTH dysregulation	Pseudohypoparathyroidism type 1A, chronic hypocalcemia
Nephrocalcinosis in treated hypocalcemia	Detected on ultrasound	Hypercalciuria	Autosomal dominant hypocalcemia type 1 overtreatment

Abbreviations: Ca^2+^, calcium ion; PTH, parathyroid hormone.

## Data Availability

No new data were created or analyzed in this study. Data sharing does not apply to this article.

## Abbreviations

The following abbreviations are used in this manuscript:

ADHD	Attention-deficit hyperactivity disorder
ADH1	Autosomal dominant hypocalcemia type 1
AHO	Albright hereditary osteodystrophy
AP2σ	Adaptor-related protein complex 2 sigma
APS-1	Autoimmune polyendocrinopathy type 1
Ca^2+^	Calcium ion
CaSR/*CASR*	Calcium-sensing receptor
cAMP	Cyclic adenosine monophosphate
CRAC	Calcium release-activated calcium
DAG	Diacylglycerol
DBP	Vitamin D binding protein
ER	Endoplasmic reticulum
FGF-23	Fibroblast growth factor-23
FHH	Familial hypocalciuric hypercalcemia
iCa	Ionized calcium
IP_3_	Inositol trisphosphate
IP_3_Rs	Inositol 1,4,5-trisphosphate receptors
NCXs	Na^+^/Ca^2+^ exchangers
NSHPT	Neonatal severe hyperparathyroidism
PHP	Pseudohypoparathyroidism
PHP1A	Pseudohypoparathyroidism type 1A
PHP1B	Pseudohypoparathyroidism type 1B
PIP_2_	Phosphatidylinositol 4,5-bisphosphate
PMCAs	Plasma membrane Ca^2+^-ATPases
PPHP	Pseudopseudohypoparathyroidism
PTH	Parathyroid hormone
RyRs	Ryanodine receptors
SERCA	Sarco/endoplasmic reticulum Ca^2+^-ATPase
SOCE	Store-operated calcium entry
STIM1	Stromal interaction molecule 1
tCa	Total calcium
TRPV6	Transient receptor potential vanilloid 6
VDDR	Vitamin D-dependent rickets
VGCCs	Voltage-gated calcium channels
25(OH)D	Total 25-hydroxyvitamin D
